# αO-Conotoxin GeXIVA[1,2] Attenuates Paclitaxel-Induced Neurotoxicity by Suppressing Ferroptosis via the Nrf2/SLC7A11/GSH/GPX4 Pathway

**DOI:** 10.3390/md24090311

**Published:** 2026-09-04

**Authors:** Dongmeng Liu, Jiaqi Yu, Weifeng Xu, Yihong Shen, Xiaoli Feng, Xiaodan Li, Sulan Luo, Jiaolin Bao, Ren-Bo Ding

**Affiliations:** 1 Key Laboratory of Tropical Biological Resources of Ministry of Education, School of Pharmaceutical Sciences, Hainan University, Haikou 570228, China; 22110710000028@hainanu.edu.cn (D.L.); 23211007000023@hainanu.edu.cn (J.Y.); 19071010110011@hainanu.edu.cn (W.X.); 24211007000028@hainanu.edu.cn (Y.S.); 22220860000095@hainanu.edu.cn (X.F.); 993586@hainanu.edu.cn (X.L.); 2 Guangxi Key Laboratory of Special Biomedicine, School of Medicine, Guangxi University, Nanning 530004, China; 3 State Key Laboratory of Mechanism and Quality of Chinese Medicine, University of Macau, Macao 999078, China

**Keywords:** GeXIVA[1,2], ferroptosis, paclitaxel, neurotoxicity, chemotherapy

## Abstract

Chemotherapy-induced neurotoxicity, affecting both the central and peripheral nervous systems, is a frequent and severe adverse effect of paclitaxel (PAC) treatment with limited therapeutic options. We previously demonstrated that PAC triggers neuronal cell death via ferroptosis. αO-Conotoxin GeXIVA[1,2], a marine-derived peptide, has shown efficacy in alleviating chemotherapy-induced neuropathic pain. In the present study, we investigated whether GeXIVA[1,2] protects neurons from PAC-induced neurotoxicity by suppressing ferroptosis. Using SH-SY5Y and HT-22 neuronal cell lines, we found that GeXIVA[1,2] pretreatment rescued PAC-impaired cell viability without exhibiting cytotoxicity. GeXIVA[1,2] markedly attenuated PAC-induced reactive oxygen species (ROS) overproduction and restored intracellular glutathione (GSH) levels. Mechanistically, PAC suppressed the Nrf2/SLC7A11/GSH/GPX4 ferroptosis-defense pathway, and GeXIVA[1,2] reactivated this axis by upregulating Nrf2, SLC7A11, and GPX4 protein expression. These results reveal a novel ferroptosis-suppressive function of GeXIVA[1,2] in the context of PAC-induced neurotoxicity. Our findings provide a mechanistic foundation for developing GeXIVA[1,2] as a ferroptosis-targeted intervention against chemotherapy-induced neurotoxicity.

## 1. Introduction

Cancer, especially breast, ovarian, and lung cancer, is a rapidly growing global burden which reduces life expectancy and has been a leading cause of death worldwide [1]. Chemotherapy, a mainstay of conventional cancer treatment, has markedly improved median survival over the past 40 years [2]. However, many chemotherapeutic regimens are associated with significant neurotoxicity, which is clinically characterized by declines in memory, processing speed, and executive function [3,4,5,6,7]. The prevalence of chemotherapy-induced cognitive impairment has risen sharply. Despite the rising prevalence of this condition, effective management strategies and clinical guidelines are still lacking.

Paclitaxel (PAC) is a microtubule-stabilizing agent belonging to the taxane class of chemotherapeutic drugs. It is frequently used as a first-line treatment for a broad range of solid tumors, including breast, ovarian, and non-small-cell lung cancer, and is classified as an essential medicine by the World Health Organization [8,9,10]. In clinical practice, PAC frequently induces both central and peripheral neurotoxicity. While chemotherapy-induced cognitive impairment (CICI, or “chemobrain”) affects 30–50% of cancer survivors [11], paclitaxel-induced peripheral neuropathy (PIPN) is even more prevalent, occurring in up to 60% of patients and representing the major dose-limiting side effect of this drug [12,13]. Notably, chemotherapeutic agents such as PAC have been reported to promote ferroptosis in various contexts [14,15,16]. In our previous study, we demonstrated for the first time that PAC-induced neuronal injury is directly mediated by ferroptosis [17]. Ferroptosis is an intracellular iron-dependent form of programmed cell death characterized by a redox state imbalance. The abnormal metabolism of iron, lipids, and amino acids, together with their interactions, constitutes the primary driver of ferroptosis. Notably, intracellular iron levels are significantly elevated in cells exposed to chemotherapeutic drugs [17,18,19,20], and disruption of iron homeostasis triggers excessive production of reactive oxygen species (ROS) [21]. The brain is particularly vulnerable to ROS because of its high content of polyunsaturated fatty acids (PUFAs), which are readily oxidized to lipid peroxides [22]. Therefore, targeting ferroptosis represents a promising therapeutic strategy for alleviating chemotherapy-induced brain injury.

**Figure 1:**
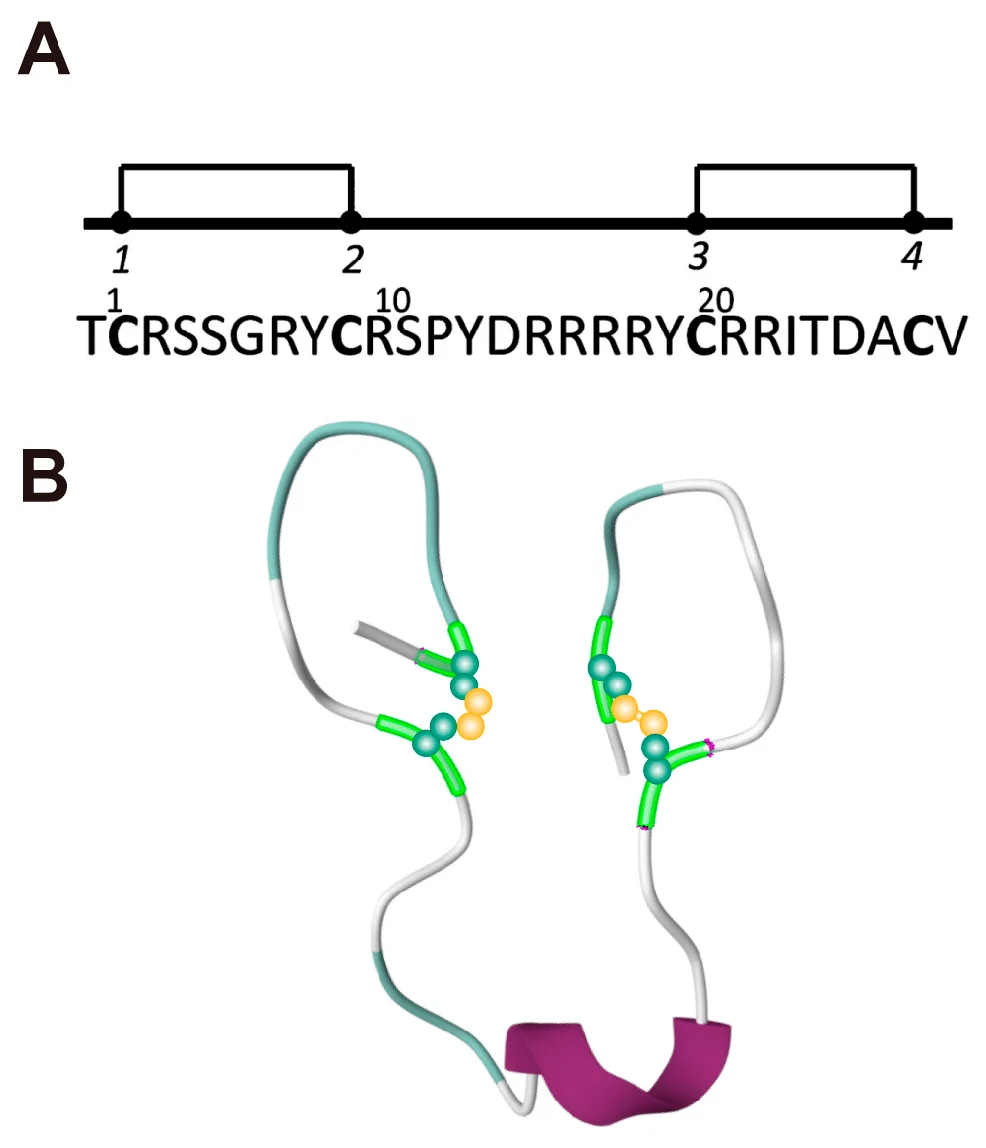
Structural features of αO-conotoxin GeXIVA[1,2]. (**A**) Amino acid sequence of GeXIVA[1,2] showing the disulfide bond connectivity (Cys2–Cys9 and Cys20–Cys27) of the bead isomer in schematic form. (**B**) Three-dimensional structural model of GeXIVA[1,2]. The disulfide bonds are shown in yellow (Cys2–Cys9 and Cys20–Cys27), and cysteine residues are shown in green.

αO-Conotoxin GeXIVA[1,2] is a 28-amino-acid peptide originally isolated from the venom of the marine cone snail *Conus generalis*. It contains four cysteine residues that form two disulfide bridges in the bead configuration, specifically between Cys2–Cys9 and Cys20–Cys27 (Figure 1A). A three-dimensional structural model of GeXIVA[1,2] is also shown, with disulfide bonds highlighted in yellow and cysteine residues in green (Figure 1B). This peptide was first discovered and synthesized by our group [23], and we previously identified it as a neuroprotective agent [24,25]. It acts as a potent and selective antagonist of the α9α10 nicotinic acetylcholine receptor (nAChR) and promotes recovery from neurotoxicity [26,27]. nAChRs are ligand-gated ion channels widely expressed in the central and peripheral nervous systems that play critical roles in the pathogenesis of neurological disorders. Among them, the α9α10 nAChR subtype has recently emerged as a promising target for neurotoxicity treatment [26,28]. Importantly, our prior work has also shown that GeXIVA[1,2] can reverse chemotherapy-induced neuropathic pain and facilitate functional recovery in vivo [24,25,29,30], highlighting its disease-modifying and neuroprotective potential. However, despite these established neuroprotective and analgesic properties, whether GeXIVA[1,2] can directly protect neurons from paclitaxel-induced ferroptosis and the signaling pathway through which this protection is mediated remain unexplored. Therefore, the present study was designed to investigate the neuroprotective effect of GeXIVA[1,2] against paclitaxel-induced ferroptosis and to elucidate the underlying molecular mechanism.

Based on our previous finding that PAC induces neuronal injury via ferroptosis [17], and on the demonstrated ability of GeXIVA[1,2] to alleviate chemotherapy-induced neuropathic pain in vivo [24,25], we hypothesized that GeXIVA[1,2] may protect neurons against PAC-induced neurotoxicity by suppressing ferroptosis. Therefore, the present study was designed to investigate the neuroprotective efficacy of GeXIVA[1,2] against paclitaxel-induced neurotoxicity and to explore the underlying mechanisms, specifically whether GeXIVA[1,2] exerts its protective effects by suppressing ferroptosis via regulation of the Nrf2/SLC7A11/GSH/GPX4 signaling pathway.

## 2. Results

### 2.1. GeXIVA[1,2] Exhibits No Cytotoxicity in SH-SY5Y and HT-22 Neuronal Cells

**Figure 2:**
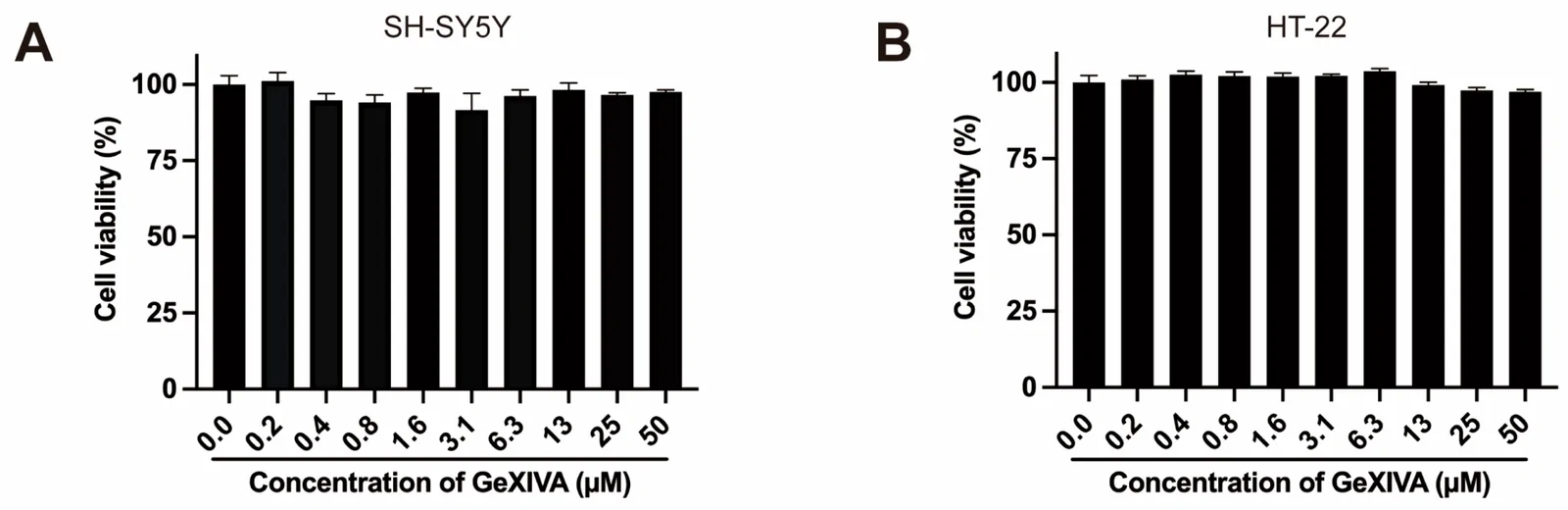
Cell viability of SH-SY5Y and HT-22 neuronal cells treated with GeXIVA[1,2]. (**A**,**B**) SH-SY5Y (**A**) and HT-22 (**B**) cells were treated with the indicated concentrations of GeXIVA[1,2] for 72 h. Cell viability was determined by MTT assay. Data are from one representative experiment (*n* = 4 biological replicates) and the experiment was independently repeated at least three times with similar results. One-way ANOVA followed by Dunnett’s post hoc test was performed.

We assessed the cytotoxic effects of GeXIVA[1,2] on neuronal cells. Treatment with GeXIVA[1,2] at concentrations ranging from 0 to 50 µM for 72 h did not cause any statistically significant reduction in cell viability in either SH-SY5Y or HT-22 cells (Figure 2). Cell viability in all GeXIVA[1,2]-treated groups remained comparable to that of the untreated control group. These results indicate that GeXIVA[1,2] does not exhibit inherent cytotoxicity in neuronal cells, providing a favorable safety profile for subsequent neuroprotective studies.

### 2.2. GeXIVA[1,2] Rescues PAC-Impaired Neuronal Viability

**Figure 3:**
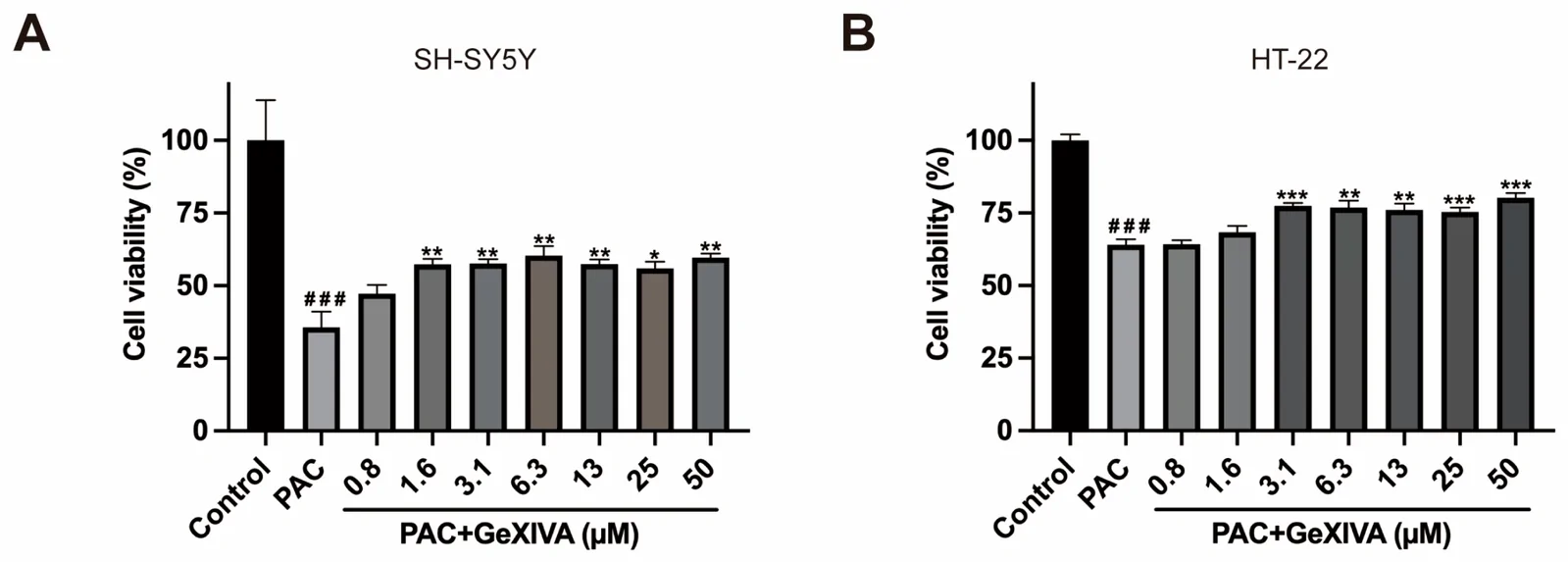
Cell viability of PAC-exposed SH-SY5Y and HT-22 neuronal cells with or without GeXIVA[1,2]. (**A**,**B**) SH-SY5Y (**A**) and HT-22 (**B**) cells were treated with PAC (30 nM and 0.25 µM, respectively) with or without a 12-h GeXIVA[1,2] pretreatment at the indicated concentrations. Cell viability was determined by MTT assay. Data are from one representative experiment (*n* = 4 biological replicates) and the experiment was independently repeated at least three times with similar results. Two-way ANOVA followed by Dunnett’s post hoc test was performed. Compared with the control group, ### *p* < 0.001; compared with the PAC group, * *p* < 0.05, ** *p* < 0.01, *** *p* < 0.001.

We next examined whether GeXIVA[1,2] could directly protect neuronal cells from PAC-induced cytotoxicity. PAC treatment resulted in a marked decline in cell viability compared with untreated controls (Figure 3A,B). Pretreatment with GeXIVA[1,2] for 12 h substantially reversed the loss of viability. In SH-SY5Y cells, significant protection was observed starting at 1.6 µM, and the protective effect was further enhanced at higher dosages (Figure 3A). A similar rescue pattern was observed in HT-22 cells (Figure 3B). These findings indicate that GeXIVA[1,2] effectively counteracts PAC-induced neuronal cell death. Based on the prominent neuroprotective efficacy observed at 13, 25, and 50 µM, these three concentrations were selected for all subsequent mechanistic studies.

### 2.3. GeXIVA[1,2] Suppresses PAC-Induced ROS Overproduction

**Figure 4:**
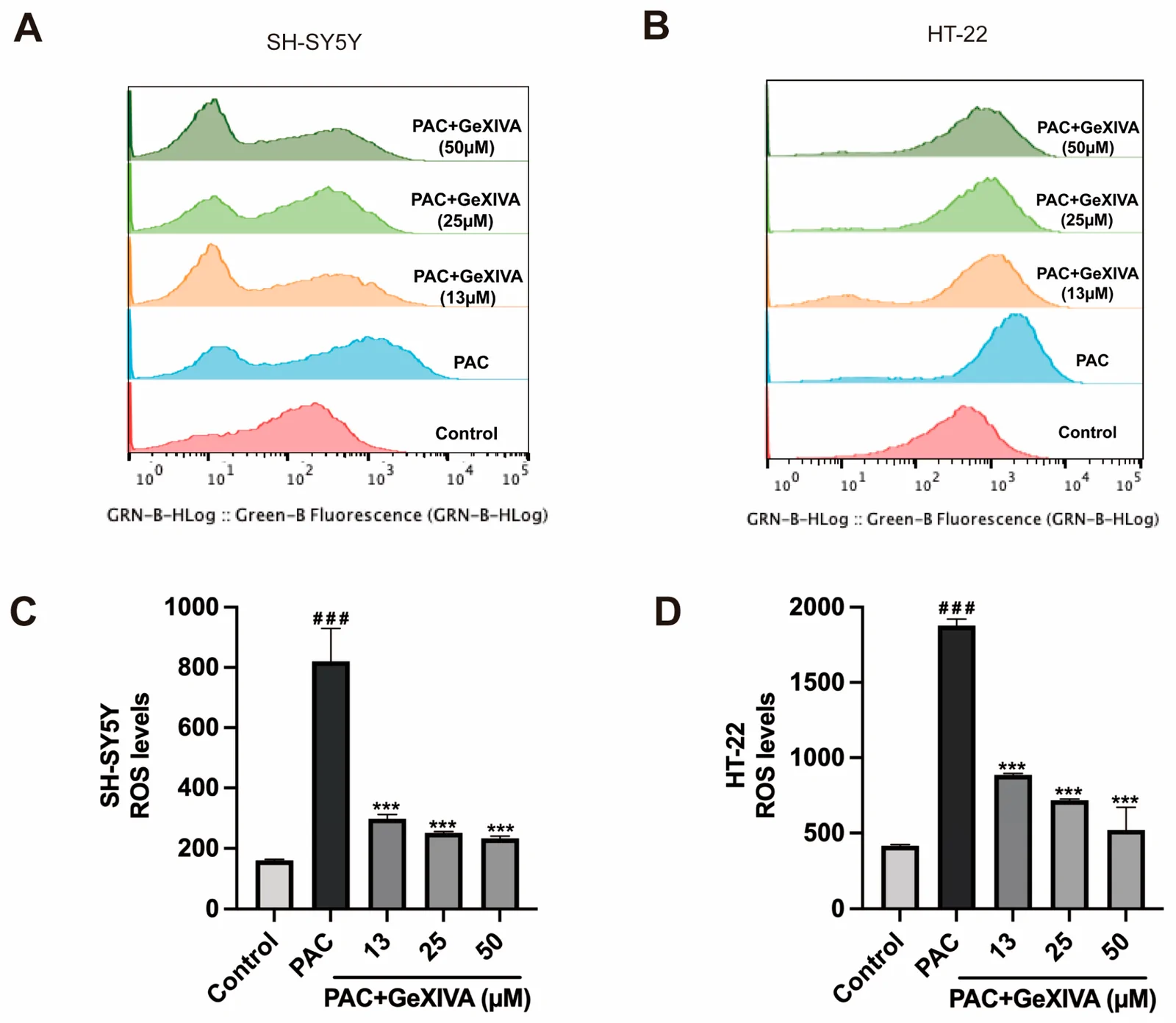
Flow cytometric analysis of ROS generation in PAC-treated SH-SY5Y and HT-22 cells with or without GeXIVA[1,2]. (**A**,**B**) Representative flow cytometry histograms of ROS levels stained with DCFH-DA in SH-SY5Y (**A**) and HT-22 (**B**) cells treated with PAC, with or without GeXIVA[1,2] pretreatment. (**C**,**D**) Quantitative analysis of mean fluorescence intensity in SH-SY5Y (**C**) and HT-22 (**D**) cells. Data are presented as mean ± SD. Data are from one representative experiment (*n* = 3 biological replicates) and the experiment was independently repeated at least three times with similar results. Two-way ANOVA followed by Dunnett’s post hoc test was performed. ### *p* < 0.001 vs. control; *** *p* < 0.001 vs. PAC group.

We examined whether GeXIVA[1,2] could attenuate PAC-induced ROS overload. PAC treatment led to a marked increase in ROS production in both cell lines (Figure 4A–D). This ROS overproduction was substantially and dose-dependently reversed by GeXIVA[1,2] at 13, 25, and 50 µM. These results indicate that GeXIVA[1,2] effectively mitigates the oxidative burst associated with PAC-induced ferroptosis.

### 2.4. GeXIVA[1,2] Restores Intracellular GSH Levels in PAC-Treated Neurons

**Figure 5:**
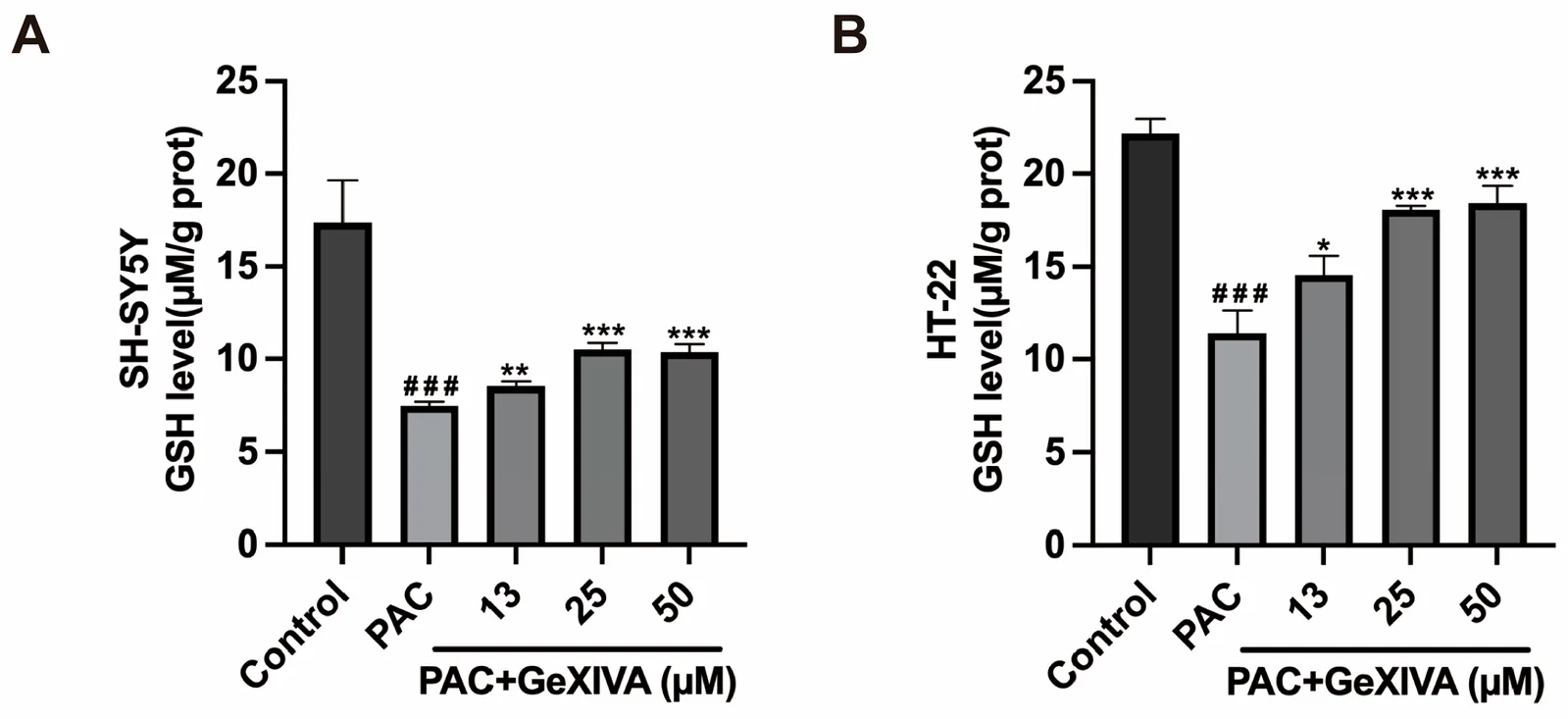
GSH level in PAC-exposed SH-SY5Y and HT-22 cells with or without GeXIVA[1,2]. (**A**,**B**) Intracellular GSH content in SH-SY5Y (**A**) and HT-22 (**B**) cells after PAC exposure with or without a 12-h GeXIVA[1,2] pretreatment. Data are mean ± SD. Data are from one representative experiment (*n* = 3 biological replicates) and the experiment was independently repeated at least three times with similar results. Two-way ANOVA followed by Dunnett’s post hoc test was performed. ### *p* < 0.001 vs. control; * *p* < 0.05, ** *p* < 0.01, *** *p* < 0.001 vs. PAC group.

We next measured intracellular GSH levels in PAC-challenged neurons. In agreement with the ROS results, PAC exposure caused a significant depletion of GSH in both SH-SY5Y and HT-22 cells (Figure 5A,B). Notably, GeXIVA[1,2] pretreatment dose-dependently restored GSH content nearly to control levels, suggesting that GeXIVA[1,2] reinforces the GSH-dependent antioxidant system to counteract ferroptosis.

### 2.5. GeXIVA[1,2] Activates the Nrf2/SLC7A11/GSH/GPX4 Signaling Pathway

**Figure 6:**
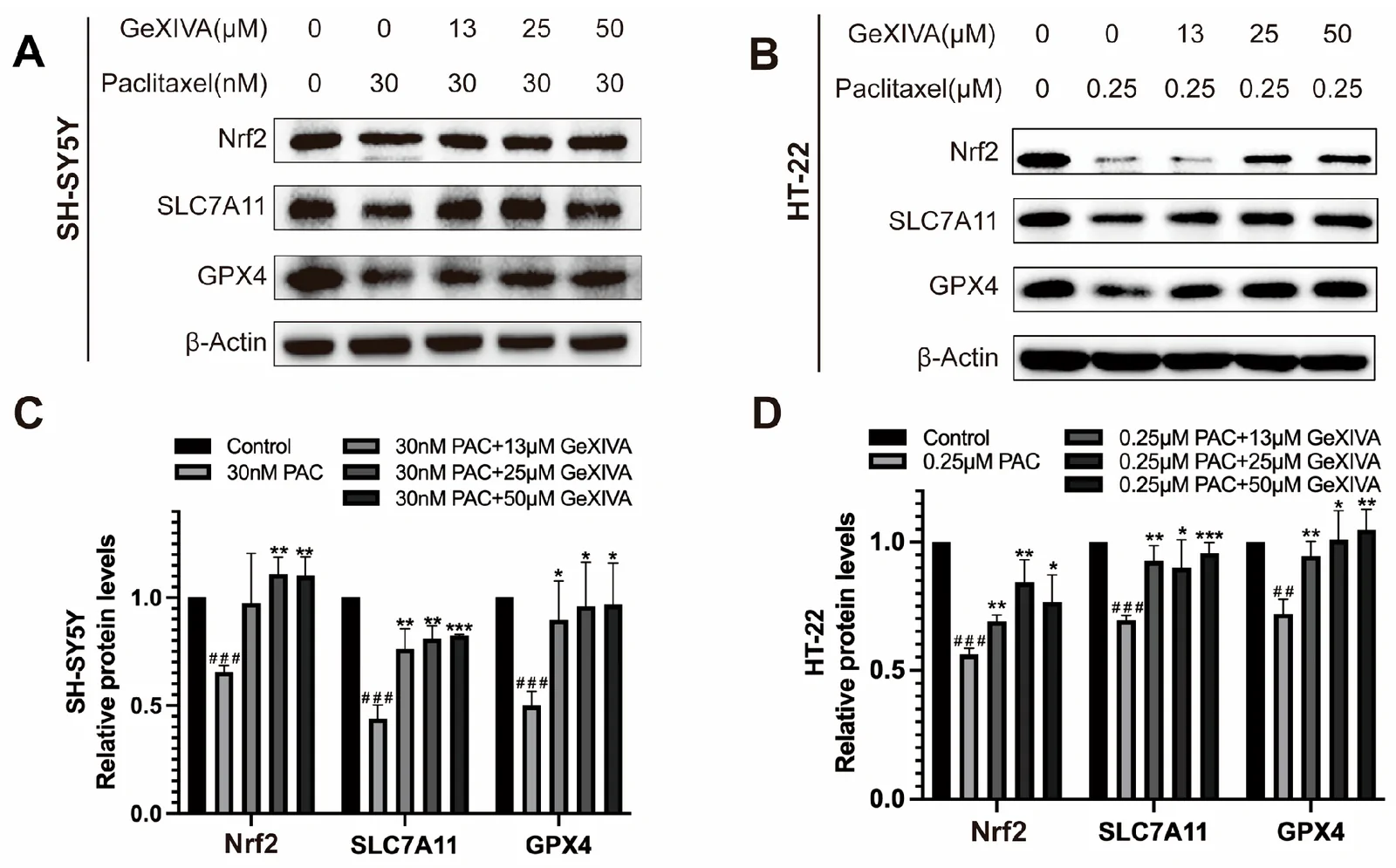
Protein expression of Nrf2, SLC7A11, and GPX4 in PAC-exposed SH-SY5Y and HT-22 cells with or without GeXIVA[1,2]. (**A**,**B**) Representative Western blot images of Nrf2, SLC7A11, and GPX4 in SH-SY5Y (**A**) and HT-22 (**B**) cells. (**C**,**D**) Densitometric quantification normalized to β-actin. Data are mean ± SD (*n* = 3 independent biological replicates). Two-way ANOVA followed by Dunnett’s post hoc test was performed. ## *p* < 0.01, ### *p* < 0.001 vs. control; * *p* < 0.05, ** *p* < 0.01, *** *p* < 0.001 vs. PAC group.

**Figure 7:**
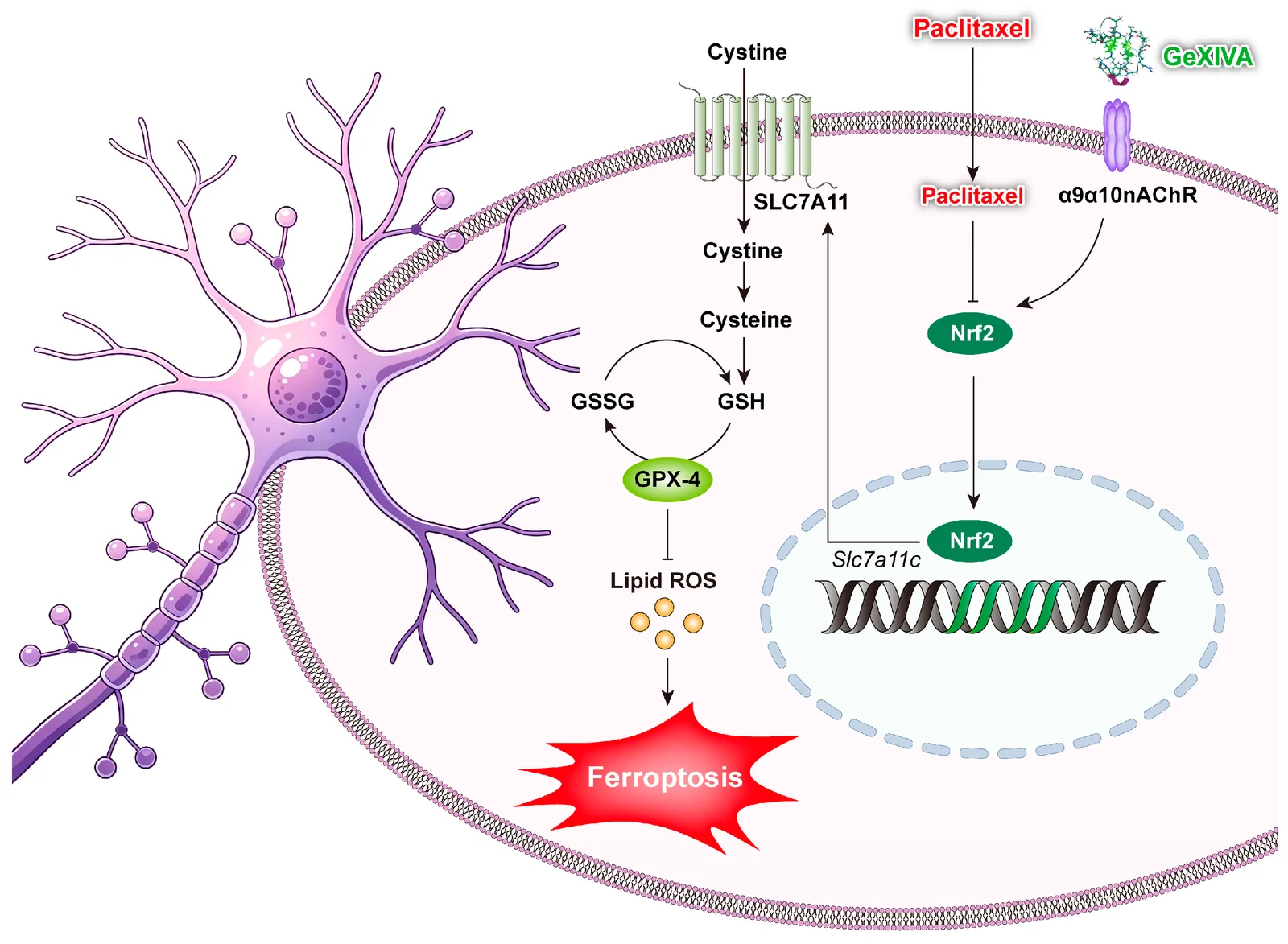
Schematic illustration of the proposed neuroprotective mechanism of GeXIVA[1,2] against paclitaxel-induced ferroptosis in neurons. Paclitaxel suppresses the Nrf2/SLC7A11/GSH/GPX4 signaling axis, leading to intracellular ROS accumulation and GSH depletion, which ultimately promotes ferroptotic neuronal cell death. GeXIVA[1,2] reactivates the Nrf2/SLC7A11/GSH/GPX4 pathway, thereby restoring GSH levels, attenuating ROS production, and protecting neurons from ferroptosis.

We examined the expression of key proteins along the Nrf2/SLC7A11/GSH/GPX4 axis in PAC-exposed cells with or without GeXIVA[1,2] pretreatment. Western blot analysis revealed that PAC treatment markedly downregulated the protein levels of Nrf2, SLC7A11, and GPX4 in both SH-SY5Y and HT-22 cells (Figure 6A–D). In contrast, GeXIVA[1,2] pretreatment significantly reversed these reductions in a dose-dependent manner. Collectively, these data demonstrate that GeXIVA[1,2] exerts neuroprotective effects against PAC-induced ferroptosis by reactivating the Nrf2/SLC7A11/GSH/GPX4 signaling pathway (Figure 7).

## 3. Discussion

The neurotoxic side effects of PAC involving both the central and peripheral nervous systems have been consistently reported. However, the mechanisms underlying PAC neurotoxicity have remained incompletely understood, limiting the development of effective interventions. Our recent work provided the first direct evidence that PAC triggers neuronal cell death via ferroptosis, and identified the Nrf2/SLC7A11/GSH/GPX4 pathway as the critical suppressed axis [17]. The present study builds on this discovery by demonstrating that αO-conotoxin GeXIVA[1,2], a peptide our group first discovered and synthesized [23], can protect neurons from PAC-induced ferroptosis by reactivating this pathway.

GeXIVA[1,2] was initially characterized as a potent and selective antagonist of the α9α10 nicotinic acetylcholine receptor (nAChR) with analgesic properties. Our previous studies in animal models have demonstrated that GeXIVA[1,2] effectively alleviates chemotherapy-induced neuropathic pain, a cardinal symptom of PIPN, and promotes functional recovery [24,25]. These findings suggested that GeXIVA[1,2] possesses neuroprotective potential in chemotherapy-induced neurotoxicity. However, whether this peptide could directly protect neurons from PAC-induced cell death, and whether such protection involves the ferroptosis pathway we identified, were unknown. The present study fills this gap.

The results presented in this study demonstrate, for the first time, that GeXIVA[1,2] protects neuronal cells from PAC-induced ferroptosis. This conclusion is supported by multiple lines of evidence. First, GeXIVA[1,2] pretreatment dose-dependently rescued cell viability in both SH-SY5Y and HT-22 neuronal lines exposed to PAC. Second, it markedly suppressed the overproduction of reactive oxygen species (ROS), a hallmark and driver of ferroptosis. Third, it restored intracellular glutathione (GSH) levels, thereby replenishing the substrate essential for GPX4-mediated lipid peroxide detoxification. Fourth, and most mechanistically informative, GeXIVA[1,2] reactivated the entire Nrf2/SLC7A11/GSH/GPX4 signaling cascade that was suppressed by PAC. The coordinated recovery of Nrf2, SLC7A11, GSH, and GPX4 strongly suggests that GeXIVA[1,2] restores the complete ferroptosis-defense relay: from Nrf2-driven transcription of SLC7A11, through SLC7A11-mediated cystine uptake and GSH biosynthesis, to GPX4-catalyzed elimination of phospholipid hydroperoxides.

These findings are consistent with the established framework of ferroptosis regulation. The brain is particularly vulnerable to ferroptosis due to its high iron content and abundance of polyunsaturated fatty acids (PUFAs) [31,32,33,34], which are readily oxidized via the Fenton reaction to generate lipid peroxides [35,36]. Under normal conditions, neurons are protected by an integrated antioxidant defense system. The transcription factor Nrf2 governs both oxidative stress responses and iron homeostasis, thereby exerting a bifunctional modulatory effect on ferroptosis [37]. Upon oxidative stress, Nrf2 translocates to the nucleus and transcriptionally activates target genes, most notably SLC7A11 [38,39]. SLC7A11, the catalytic subunit of the cystine/glutamate antiporter system xc^−^ [40], mediates cystine uptake to sustain GSH biosynthesis [41,42]. GSH not only functions as a major cellular antioxidant [43] but also serves as the obligatory substrate for GPX4 [44], the central enzyme that catalyzes the reduction of phospholipid hydroperoxides to inert alcohols, thereby restraining ferroptosis [45,46]. Our data demonstrate that PAC simultaneously disrupts multiple nodes of this defense network, and that GeXIVA[1,2] is capable of partially restoring the activity of this pathway.

The demonstration that an α9α10 nAChR antagonist can regulate ferroptosis suggests a novel crosstalk between cholinergic signaling and iron/redox metabolism in neurons. The α9α10 nicotinic acetylcholine receptor (nAChR) has recently emerged as a promising therapeutic target for neuropathic pain. Specific antagonists of this receptor, notably several conotoxins, not only alleviate neuropathic symptoms but also promote neural lesion repair, suggesting a disease-modifying potential [26,27,28]. Previous studies have demonstrated that α9α10 nAChR antagonists, including RgIA analogs and oligoarginine R8, effectively alleviate both acute and long-term chemotherapy-induced neurotoxicity [47,48,49,50]. Our previous work has established GeXIVA[1,2] as a potent and selective α9α10 nAChR antagonist with analgesic and neurorestorative properties [24,25]. The present findings considerably extend its pharmacological profile by linking α9α10 nAChR blockade to Nrf2-mediated ferroptosis defense. This dual functionality positions GeXIVA[1,2] as a multi-target agent capable of simultaneously addressing neuropathic pain and neuronal ferroptosis, two interrelated pathologies in PAC neurotoxicity. The mechanistic connection we uncovered—that antagonizing α9α10 nAChR leads to Nrf2 activation and subsequent upregulation of the SLC7A11/GSH/GPX4 axis—suggests a previously unrecognized crosstalk between cholinergic signaling and iron/redox metabolism in neurons. Whether this crosstalk is direct (e.g., via nAChR-mediated calcium signaling that modulates Nrf2 stability) or indirect remains an important question for future investigation.

The involvement of ferroptosis in peripheral neuropathy is an emerging concept. Our previous finding that PAC suppresses the Nrf2/SLC7A11/GSH/GPX4 pathway [17], together with the present demonstration that restoring this pathway with GeXIVA[1,2] prevents neuronal ferroptosis, provides a compelling mechanistic framework for understanding and potentially treating both central and peripheral PAC neurotoxicity. The coordinated attack on sensory signaling (via α9α10 nAChR antagonism) and ferroptotic death (via Nrf2 pathway reactivation) may offer synergistic benefits that single-target approaches cannot achieve. The ability of GeXIVA[1,2] to simultaneously target α9α10 nAChR-mediated pain signaling and ferroptosis execution positions it as a unique dual-action candidate for combating both the sensory and degenerative components of PAC neurotoxicity. Future studies using primary dorsal root ganglion neurons, iPSC-derived peripheral sensory neurons, and in vivo CICI/PIPN models will be essential to validate the translational relevance of ferroptosis inhibition in the peripheral nervous system.

We acknowledge several limitations of the current study. The use of monocultured SH-SY5Y and HT-22 cell lines, while providing a well-controlled platform for mechanistic dissection, does not capture the complexity of the nervous system microenvironment, including neuron–glia interactions and systemic metabolic influences. Furthermore, whether GeXIVA[1,2] can effectively cross the blood–nerve barrier and blood–brain barrier, or requires alternative delivery strategies, needs to be addressed. In addition, the concentrations of GeXIVA[1,2] selected for mechanistic assays (13–50 µM) were chosen to ensure robust and reproducible detection of pathway changes; however, lower concentrations also showed protective trends in the viability assay. Future dose–response optimization studies will be important to define the minimal effective concentration and therapeutic window of GeXIVA[1,2]. Finally, although we established the necessity of the Nrf2/SLC7A11/GSH/GPX4 pathway for the neuroprotective effect of GeXIVA[1,2] through pharmacological correlation, direct causal evidence using Nrf2 knockout or SLC7A11 knockdown approaches would further strengthen the mechanistic conclusions. These questions represent important directions for our ongoing and future work.

## 4. Materials and Methods

### 4.1. Cell Culture and Pharmacological Treatments

Human SH-SY5Y and murine HT-22, obtained from National Collection of Authenticated Cell Cultures (Shanghai, China), were kept in a humidified incubator at 37 °C with 5% CO_2_. SH-SY5Y cells were maintained in specialized complete medium (CM-0208, Procell, Wuhan, China), whereas HT-22 cells were cultured in DMEM medium (PM150210, Procell, Wuhan, China) supplemented with 10% fetal bovine serum (164210, Procell, Wuhan, China) and 1% penicillin/streptomycin (PB180120, Procell, Wuhan, China). To establish a PAC-induced neurotoxicity model, cells were exposed to PAC (P815862, Macklin, Shanghai, China) at concentrations of 30 nM (for SH-SY5Y) and 0.25 µM (for HT-22) for 72 h, according to the optimized conditions derived from our previous work. For the intervention group, cells were pretreated with GeXIVA[1,2] for 12 h prior to PAC administration.

### 4.2. Peptide Synthesis

The synthesis and purification of GeXIVA[1,2] were performed as previously reported [23,51]. Briefly, the linear peptide with protected side chains was synthesized via Fmoc solid-phase peptide synthesis chemistry. Subsequently, a two-step oxidation approach was employed to form the disulfide bridges [52]. Finally, liquid chromatography–mass spectrometry (LCMS) and ultra-performance liquid chromatography (UPLC) were used to verify the molecular weight and purity of the compound, respectively [23].

### 4.3. MTT Assay

Cell viability was determined via the MTT assay (ST1537, Beyotime, Shanghai, China). Briefly, cells were plated in 96-well plates at a density of 5,000 cells per well and allowed to adhere overnight. To evaluate the potential neurotoxicity of GeXIVA[1,2] itself, SH-SY5Y (human neuroblastoma) and HT-22 (mouse hippocampal neurons) cells were exposed to increasing concentrations of GeXIVA[1,2] (0–50 µM) for 72 h. For the viability rescue assay, SH-SY5Y and HT-22 cells were exposed to PAC at the predetermined neurotoxic concentrations (30 nM and 0.25 µM, respectively). Each experimental group included four biological replicate wells, and the entire experiment was independently repeated at least three times with similar results. Following the 72-h PAC exposure (with or without GeXIVA[1,2] pretreatment), 5 mg/mL MTT reagent was added to each well, and the plates were incubated for an additional 4 h to enable formazan crystal formation. After careful removal of the supernatant, the formazan precipitates were solubilized in 100 µL of dimethyl sulfoxide (DMSO). The optical density was recorded at 570 nm using a microplate reader, and the absorbance values were used to calculate the percentage of viable cells relative to controls.

### 4.4. Measurement of Intracellular ROS

Intracellular ROS levels were measured by flow cytometry using a commercial assay kit (S0033S, Beyotime, Shanghai, China) according to the manufacturer’s protocol. Briefly, cells were stained with 10 µM DCFH-DA for 20 min at 37 °C in the dark. The cells were then collected, washed twice with PBS, and resuspended for flow cytometric analysis. Each experimental group included three biological replicates, and the entire experiment was independently repeated at least three times with similar results. Fluorescence intensity was recorded for at least 10,000 events per sample, and the mean fluorescence intensity was used to determine relative ROS production.

### 4.5. Detection of Glutathione (GSH) Level

Intracellular GSH levels were evaluated using a commercial GSH assay kit (A006-2-1, Nanjing Jiancheng Bioengineering Institute, Nanjing, China) according to the manufacturer’s instructions. Following the indicated treatments, cells were collected, washed twice with ice-cold PBS, and lysed to obtain the supernatant for GSH measurement. Each experimental group included three biological replicates, and the entire experiment was independently repeated at least three times with similar results. The absorbance of the reaction product was measured at 420 nm using a microplate reader, and the GSH content was determined from a standard curve and normalized to the total protein concentration of each sample.

### 4.6. Western Blot Analysis

To evaluate protein expression levels, cells were collected after the indicated treatments and lysed in radioimmunoprecipitation assay (RIPA) lysis buffer (P0013J, Beyotime, Shanghai, China) supplemented with a protease inhibitor cocktail to maintain protein integrity. Lysates were cleared by centrifugation at 12,000× *g* for 15 min at 4 °C, and the supernatants were collected. The protein concentration of each lysate was determined using the bicinchoninic acid (BCA) Protein Assay Kit (Beyotime, Shanghai, China). Equal amounts of protein (20 µg per lane) were separated by sodium dodecyl sulfate–polyacrylamide gel electrophoresis (SDS-PAGE) and subsequently electrotransferred onto polyvinylidene difluoride (PVDF) membranes (SEQ00010, Merck Millipore, Darmstadt, Germany). The membranes were blocked with 5% non-fat milk or bovine serum albumin (BSA) in Tris-buffered saline containing Tween-20 (TBST) for 1 h at room temperature to prevent non-specific binding. Thereafter, the membranes were incubated overnight at 4 °C with primary antibodies against nuclear factor-E2-related factor 2 (Nrf2) (1:1000; AF0639; Affinity Biosciences, Changzhou, China), glutathione peroxidase 4 (GPX4) (1:1000; 67763-1-Ig; Proteintech, Wuhan, China), solute carrier family 7 member 11 (SLC7A11) (1:1000; DF12509; Affinity Biosciences), and β-actin (1:5000; AF7018; Affinity Biosciences). After washing with TBST, the membranes were incubated with horseradish peroxidase (HRP)-conjugated secondary antibodies, including anti-mouse (1:5000; SA00001-1; Proteintech) and anti-rabbit (1:5000; SA00001-2; Proteintech), for 1 h at room temperature. Protein bands were visualized using an enhanced chemiluminescence (ECL) detection system (WBKLS0500, Merck Millipore, Darmstadt, Germany) and imaged with a chemiluminescence imaging system. Band intensities were quantified using ImageJ software (version 1.53k, National Institutes of Health, Bethesda, MD, USA) and normalized to the corresponding β-actin loading control.

### 4.7. Statistical Analysis

All experiments were performed at least three independent times, and data are represented as mean ± SD. In the figure legends, “n” refers to the number of biological replicates within a representative experiment; for Western blot, “n” represents independent experiments. All statistical analyses were conducted using SPSS 25.0 statistical software. Normality of data distribution was assessed using the Shapiro–Wilk test, and homogeneity of variances was evaluated using Levene’s test. For comparisons involving more than two groups, one-way or two-way analysis of variance (ANOVA) was used, followed by Dunnett’s post hoc test for multiple comparisons. Statistical significance was defined as *p* < 0.05.

## 5. Conclusions

In summary, this study demonstrates that αO-conotoxin GeXIVA[1,2] effectively protects neuronal cells from paclitaxel-induced neurotoxicity by suppressing ferroptosis. Mechanistically, GeXIVA[1,2] reactivates the Nrf2/SLC7A11/GSH/GPX4 signaling axis that is suppressed by paclitaxel, thereby attenuating ROS accumulation, restoring GSH levels, and ultimately preventing ferroptotic cell death (Figure 7). These findings reveal a novel ferroptosis-suppressive function of GeXIVA[1,2] in the context of paclitaxel-induced neurotoxicity, and uncover a previously unrecognized crosstalk between α9α10 nAChR antagonism and iron/redox metabolism. Our results provide a mechanistic foundation for developing GeXIVA[1,2] as a ferroptosis-targeted intervention against chemotherapy-induced central and peripheral neurotoxicity.

## Data Availability

Data will be made available upon request.
